# Establishing the concept of aza-[3 + 3] annulations using enones as a key expansion of this unified strategy in alkaloid synthesis

**DOI:** 10.3762/bjoc.9.131

**Published:** 2013-06-18

**Authors:** Aleksey I Gerasyuto, Zhi-Xiong Ma, Grant S Buchanan, Richard P Hsung

**Affiliations:** 1 Division of Pharmaceutical Sciences, School of Pharmacy, and Department of Chemistry, University of Wisconsin, Madison, WI 53705, USA

**Keywords:** alkaloids synthesis, catalysis, enones, intramolecular aza*-*[3 + 3] annulation, *N-*heterocycles, natural product, vinylogous amides

## Abstract

A successful enone version of an intramolecular aza-[3 + 3] annulation reaction is described here. Use of piperidinium trifluoroacetate salt as the catalyst and toluene as the solvent appears to be critical for a successful annulation. We also demonstrated for the first time that microwave irradiation can accelerate aza-[3 + 3] annulation reactions. An attempt to expand the scope of the enone aza-[3 + 3] annulation was made in the form of propyleine synthesis as a proof of concept. While synthesis of the enone annulation precursor was successfully accomplished, the annulation proved to be challenging and was only modestly successful.

## Introduction

**Figure 1:**
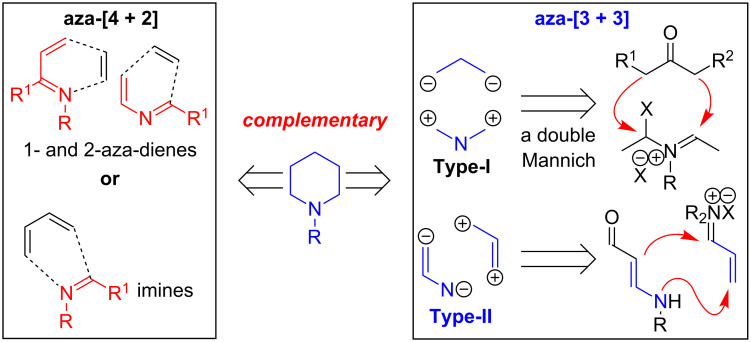
An aza-[3 + 3] annulation.

Throughout the past decade, we have been developing an aza-[3 + 3] annulation reaction as a general and unified strategy in alkaloid synthesis [1–32]. Our aza-[3 + 3] annulation, which has been classified as Type-II [1], with Type-I aza-[3 + 3] annulation being reserved for Robinson’s double Mannich-type process [33], utilizes readily accessible and easily handled vinylogous amides and vinyl iminium salts. It provides a significant complementary, if not superior, approach to aza-[4 + 2] cycloadditions in constructing piperidines, because the aza-dienes and imines required are not always the most accessible and/or easily handled substrates given the problems of isomerization and hydrolysis (Figure 1) [34–37].

**Scheme 1:**
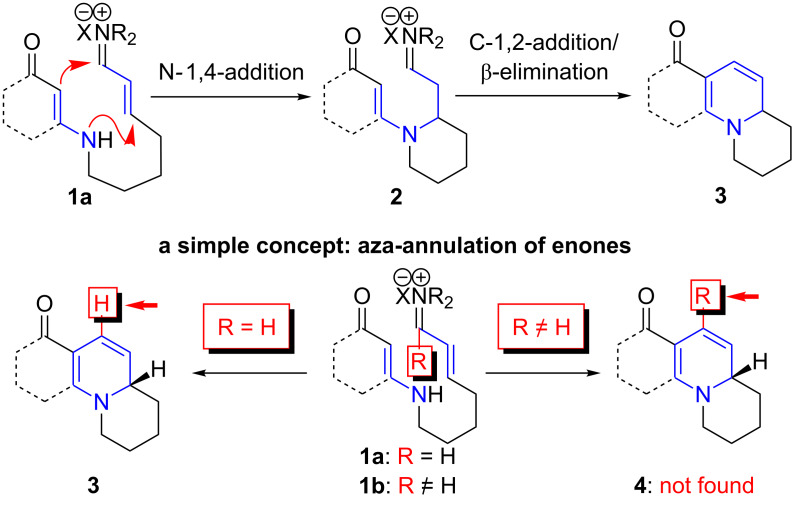
Aza-[3 + 3] annulations with enones.

The prevalence of six-membered nitrogen heterocyclic motifs in alkaloids renders the development of this aza-[3 + 3] annulation into a powerful strategy a unique opportunity in the field of alkaloid synthesis [1–4,8–15]. The intramolecular variant of this annulation has proven to be particularly valuable for total synthesis [16,26–32]. Specifically, the intramolecular aza-[3 + 3] annulation of vinylogous amides tethered to a vinyl iminium motif **1a** proceeds through a tandem sequence of N-1,4-addition and C-1,2-addition/β-elimination [16–17], effectively leading to a variety of nitrogen heterocyclic motifs, such as **3**, which are prevalent among alkaloids (Scheme 1) [16,26–32]. However, there was a significant deficiency in this annulation: the inability to employ vinylogous amides tethered to α,β-unsaturated ketones, or enones **1b** (R ≠ H) (Scheme 1).

**Figure 2:**
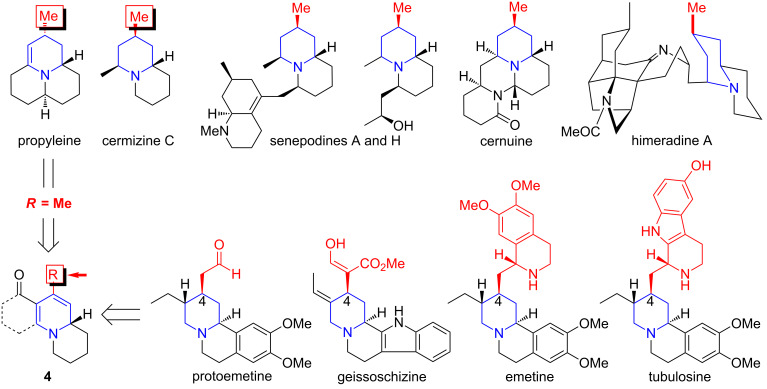
Possible natural-product targets.

Conceptually, employing enals or enones in an aza-[3 + 3] annulation appears to constitute very little difference, with the major difference lying in the R group in their respective products **3** and **4**. While seemingly an insignificant perturbation, the ability to employ enones in aza-[3 + 3] annulations can generate a vast array of opportunities, including the case where R is as small and simple as a Me group, which would already represent a facile entry to aza-phenylene alkaloids [30–31,38–45], such as propyleine [46–48], as well as lycopodium alkaloids that are rich in bioactivities [49–51] (Figure 2). In addition, the importance of these ventures into an aza-[3 + 3] annulation with enones can also be further demonstrated through syntheses of tetrahydroisoquinolines and indole alkaloids [52–55] in which the R group can constitute a larger functionality of the alkaloids, such as that seen in geissoschizine [52–57], and ipecac alkaloids such as protoemetine [48,52–55,58–61], emetine [52–55,58–63], and tubulosine [64]. With the appropriate choice of an R group in the synthetic design, a highly convergent and expedient strategy can come forth for constructing these alkaloids. We report herein our success in achieving intramolecular aza-[3 + 3] annulations of an enone.

## Results and Discussion

**Scheme 2:**
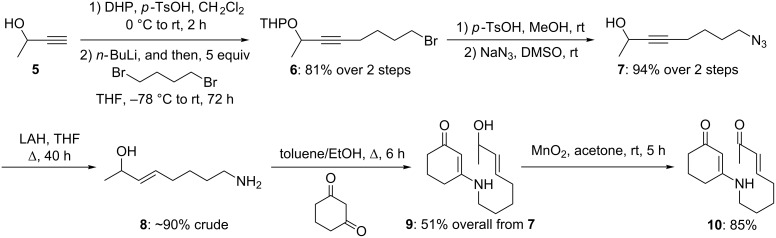
Synthesis of the annulation precursor enone **10**.

To examine the feasibility of an enone intramolecular aza-[3 + 3] annulation, a seven-step synthesis of the annulation substrate **10** commencing from 3-butyn-2-ol (**5**) was carried out (Scheme 2). Protection of secondary propargyl alcohol **5** as the THP-ether followed by alkylation of a lithium acetylide onto 1,4-dibromobutane afforded bromide **6** in 81% overall yield. Acid-mediated removal of THP followed by azide formation using NaN_3_ afforded alkyl azide **7** in 94% overall yield.

Preparation of vinylogous amide **9** was then achieved by a two-step sequence: (a) LAH-mediated azide reduction to give a primary amine with concomitant reduction of the alkyne revealing the olefin functionality; and (b) dehydrative condensation of this primary amine with 1,3-cyclohexanedione. Oxidation of the secondary allylic alcohol in **9** with MnO_2_ at ambient temperature provided enone **10** in 85% yield. We were poised to investigate the viability of the enone aza-[3 + 3] annulation.

Our initial efforts to employ enone substrate **10** met with failure when using up to 500 mol % of piperidinium chloride [65] (Table 1, entry 1) or 100 mol % piperidinium acetate salts (Table 1, entry 2); even at higher temperatures of 120 °C, these chloride and acetate salts proved to be ineffective (Table 1, entry 3). We then explored salts prepared from stronger acids such as (+)-camphorsulfonic acid (CSA) and trifluoroacetic acid, which should tend to dissociate easier in the reaction medium and lead to a more reactive vinyl iminium salt intermediate **1b**. We were elated to observe that the use of 100 mol % of piperidinium CSA salt in toluene at 120 °C provided 100% conversion with 60% yield of cycloadduct **11** (Table 1, entry 4). Further optimizations using trifluoroacetate salt (Table 1, entries 5–8) revealed that the yield could be improved to 87%, while lowering the catalyst loading from 500 mol % to 50 mol %, although the reaction temperature was raised to 150 °C. Lastly, we also examined the use of microwave irradiation [66–67] and observed a distinct rate enhancement (Table 1, entry 9).

**Table 1:** Catalyst screening and optimization.

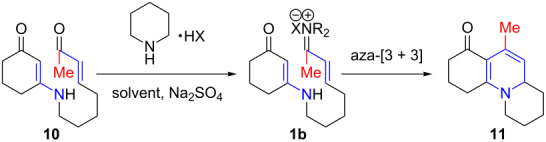						

entry^a^	HX (mol %)	solvent	temp (°C)	time (h)	conv. (%)	yield (%)^b^

1	HCl (500)	EtOAc/toluene	100	16	0	–
2	HOAc (100)	EtOAc	85	12	0	–
3	HOAc (500)	toluene	120	16	0	–
4	(+)-CSA^c^ (100)	toluene	120	12	100	60
5	HO_2_CCF_3_ (500)	toluene	85	7	79	39
6	HO_2_CCF_3_ (100)	toluene	85	5	100	50
7	HO_2_CCF_3_ (100)	EtOAc/toluene	110	4	92	41
8	HO_2_CCF_3_ (50)	toluene	150	3	100	87
9	HO_2_CCF_3_ (90)	toluene	MW^d^	1.5	94	65

^a^All reactions run in a sealed tube. Concn = 0.03–0.04 M; 5.0 equiv Na_2_SO_4_. ^b^Isolated yields. ^c^(+)-Camphorusulfonic acid. ^d^450-W Microwave.

The observed reactivity difference of the trifluoroacetate and camphorsulfonic acid salts, as compared to the chloride and acetate salts, can be attributed to the rate at which they promote vinyl iminium ion formation through a “balanced act” [12]. The difference in reactivity is related to the dissociation capacity of the respective amine salts. Since both “free amine” and “free acid” are needed in a synergistic manner to generate the vinyl iminium ion from the corresponding enone, the ability of the amine salt to dissociate to its “free amine” and “free acid” can exert an impact on the rate of the iminium formation. The low reactivity of the chloride salt can be attributed to its higher resistance toward dissociation, or is simply a tighter ion-pair compared to the CSA and the trifluoroacetate salt. At the same time, the formation of vinyl iminium ion from carbonyl systems and the “free amine” is also promoted by protonation of the carbonyl group via the “free acid”. Consequently, the increased reactivity of the CSA and trifluoroacetate salt from the acetate salt can be attributed to a higher acidity of the acids.

**Scheme 3:**
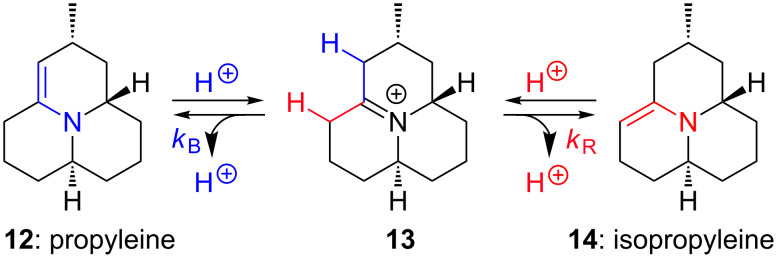
Propyleine-isopropeleine interconversion.

After establishing the feasibility of the enone version of the intramolecular aza-[3 + 3] annulation we turned our attention to propyleine (**12**) (see Figure 2 and Scheme 3) as a possible target to showcase the new enone aza-[3 + 3] annulation. Propyleine (**12**) was isolated in 1972 from *Propylaea quatuordecimpunctata* in a continued effort by Tursch and co-workers [46–47] in their isolation of the azaphenalene family of defensive alkaloids from various ladybug beetles [38–45]. Mueller and Thompson [48] in 1980 found it interesting that the isomeric enamine named isopropyleine (**14**) was not reported in the original paper [46–47] taking into account that the isolation conditions involved acid–base extractions. It is conceivable that propyleine (**12**) and isopropyleine (**14**) could be interconvertable under acidic conditions via intermediate iminium salt **13**.

To investigate this matter, Mueller and Thompson carried out the first and the only synthesis of propyleine known to date [48]. They were able to take a mixture of propyleine and isopropyleine with 1:3 ratio as determined by ^1^H NMR and watch two sets of proton resonances collapse into one that corresponds to the iminium salt **13** after addition of TFA. This experiment strongly suggested that propyleine (**12**) and isopropyleine (**14**) could equilibrate under acidic conditions, thereby implying that the observed ratio of **12** and **14** represents a thermodynamic one in favor of the more stable isopropyleine [48]. With these experimental findings, Mueller and Thompson concluded that the alkaloid isolated by Tursch and co-workers [46–47] was in fact a mixture of readily interconvertable **12** and **14**.

**Figure 3:**
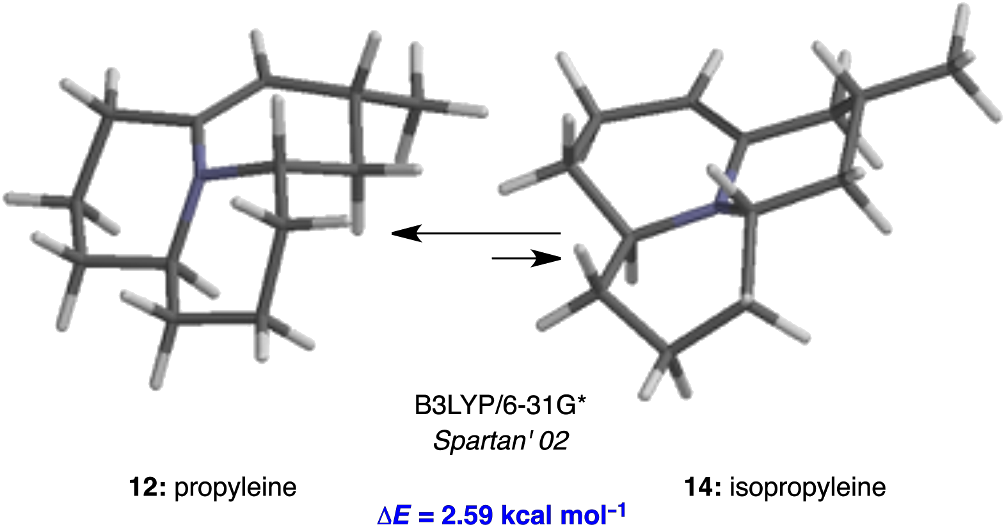
Relative stabilities of propyleine and isopropyleine.

We found this controversy in the isolation and total synthesis papers an interesting one that deserves further investigations. Consequently, we performed ab initio calculations on **12** and **14** to gain insight into their relative thermodynamic stability. Models of the most stable conformers and their corresponding energies are shown in Figure 3. To our surprise, propyleine (**12**) is 2.59 kcal mol^−1^ more stable than isopropyleine (**14**), which is the opposite of the Mueller–Thompson postulation [48]. Our calculations suggest that the ratio obtained from the Mueller–Thompson study was likely determined by the kinetics (*k*_B_ versus *k*_R_) in the deprotonation step or the tautormerization process from the iminium salt **13**, and not by thermodynamics as originally proposed. Resolving this interesting literature controversy added extra incentive for us to pursue propyleine.

**Scheme 4:**
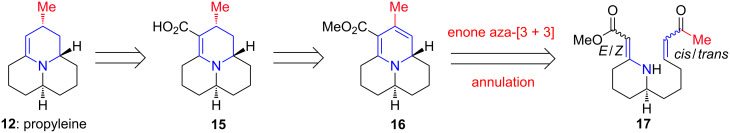
Retrosynthesis of propyleine (**12**).

Retrosynthetically, we envisioned propyleine (**12**) to come from the decarboxylation reaction of vinylogous carbamic acid **15** [10,28], which could be derived from stereoselective hydrogenation of the endocyclic olefin in tricycle **16** (Scheme 4). Vinylogous urethane **16** should be accessible via our intramolecular aza-[3 + 3] annulation of enone **17**. Enone **17** could be *E* or *Z* with respect to the vinylogous urethane olefin, and *cis* or *trans* with respect to the enone double bond. Thus, from the spectroscopic analysis stand point, synthesis of **17** could be messy.

**Scheme 5:**
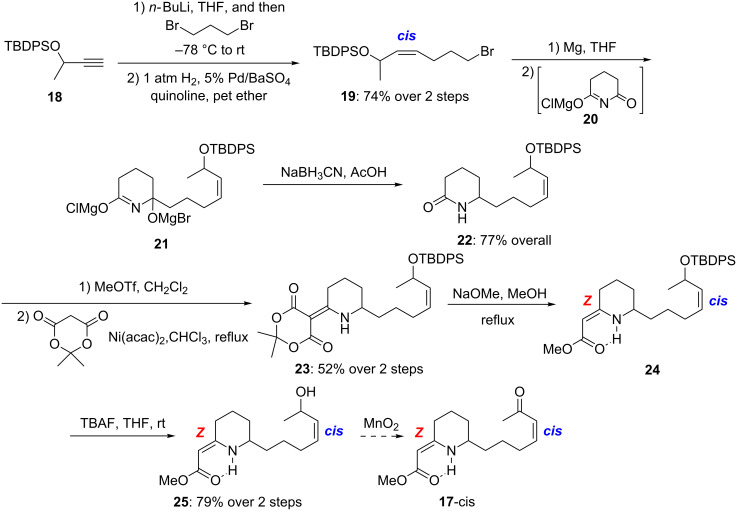
Synthesis of allyl alcohol **25**.

In the forward direction to synthesize **17**, we used the approach developed in our model study for the synthesis of precoccinelline, hippodamine and myrrhine alkaloids [30–31]. Specifically, TBDPS-protected 3-butyne-2-ol **18** was converted to bromoalkene **19** in two steps involving alkylation with an excess of dibromopropane and subsequent Lindlar’s hydrogenation (Scheme 5). Grignard addition to the glutarimide Mg-salt **20** followed by reduction of the Mg salt **21** afforded lactam **22** as an inseparable 1:1 mixture of two diastereomers in 77% overall yield [68–70].

Vinylogous amide **23** was prepared by *O*-methylation of lactam **22** with freshly distilled MeOTf followed by condensation with Meldrum’s acid in the presence of Ni(acac)_2_. Treatment of **23** with MeONa in MeOH under reflux gave vinylogous urethane **24**. It is noteworthy that the geometry of the vinylogous urethane double bond exclusively favored *Z*, presumably due to the internal hydrogen bonding. Cleavage of the TBDPS group with TBAF provided allylic alcohol **25** in 79% from **23**. However, submission of **25** to the standard MnO_2_ oxidation procedure did not lead to enone **17**, with only the starting material being recovered (Table 2). After extensive screening of various oxidation protocols, we succeeded with the Doering–Parikh conditions [71], and the *cis* geometry of the enone olefin was preserved under these conditions.

**Table 2:** Synthesis of the annulation precursor **17**.

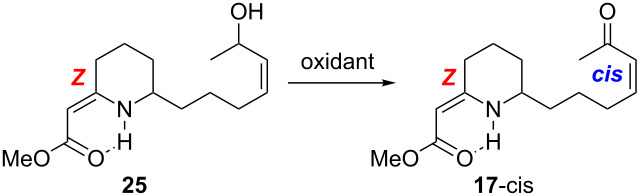			

entry	oxidants	solvent	yield (%)

1	MnO_2_	CH_2_Cl_2_	no rxn (rt)
2	MnO_2_	CH_2_Cl_2_	decomp (40 °C)
3	BaMnO_4_	CH_2_Cl_2_	no rxn
4	DMP	CH_2_Cl_2_	slow decomp
5	PCC	CH_2_Cl_2_	decomp
6	TEMPO/oxone	toluene	no rxn
7	TPAP/NMO	CH_2_Cl_2_	decomp
8	Pyr-SO_3_/DMSO	CH_2_Cl_2_	85%

With enone **17**-*cis* in hand, we studied its intramolecular aza-[3 + 3] annulation reaction utilizing piperidinium salts (Table 3). When compound **17**-*cis* was treated with the acetate salt in EtOAc at rt, no reaction was observed after 18 h (Table 3, entry 1). Heating this reaction mixture at 85 °C for 12 h only led to slow isomerization of *cis*-enone **17** to the thermodynamically more stable *trans*-isomer again with no formation of the desired annulation product **16** (Table 3, entry 2). The inefficiency of the piperidinium acetate salt in the intramolecular *aza*-[3 + 3] annulation of enones was consistent with our previous findings (see Table 1). We were also not successful in converting enone **17**-*cis* to the desired tricycle **16** using the more reactive trifluoroacetate salt and EtOAc as the solvent. In this case also only isomerization to *trans*-enone was detected (Table 3, entry 3). Heating this reaction to 130 °C led to eventual decomposition of the starting material (Table 3, entry 4).

**Table 3:** Aza-[3 + 3] annulations of **17**-*cis*.

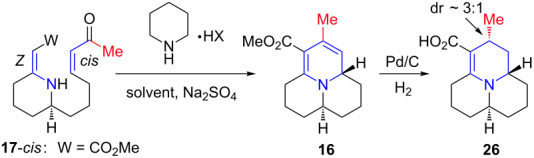					

entry^a^	HX (1.0 equiv)	solvent	temp (°C)	time (h)	yield (%)^b^

1	HOAc	EtOAc	25	18	–
2	HOAc	EtOAc	85	12	**17**-*trans*
3	HO_2_CCF_3_	EtOAc	55	12	**17**-*trans*
4	HO_2_CCF_3_	EtOAc	130	12	decomp
5	HO_2_CCF_3_	toluene	100	5	30% of **26**

^a^All reactions run in a sealed tube. Concn = 0.03–0.04 M; 5.0 equiv Na_2_SO_4_. ^b^Isolated yields.

However, when enone **17**-*cis* was heated in toluene at 100 °C, complete consumption of starting material was observed after 5 h (Table 3, entry 5). Even though the reaction was relatively messy, the formation of the desired cycloadduct **16** was confirmed by the presence of characteristic signals in the ^1^H NMR spectra of the crude mixture. Unfortunately, attempts to isolate the annulation product in pure form were not successful, probably due to the high instability of the electron-rich dihydropyridine moiety in **16**. Based on our previous experience with precarious annulation products, in situ hydrogenation was then carried out, and we managed to track down ~30% of the desired reduced aza*-*annulation product **26**, although stereoselectivity for the reduction was modest. We ultimately elected not to force our way toward propyleine using the enone aza-[3 + 3] annulation, as we succeeded in total syntheses of other members of the azaphenalene alkaloid family through annulations with enals [30–31]. While it is disappointing that this particular system may have lacked sufficient stability for this to be a suitable synthetic approach, success in an enone version of intramolecular aza-[3 + 3] annulation will allow us to find future applications.

## Conclusion

Herein, we have described a successful enone version of intramolecular aza-[3 + 3] annulation reaction. Use of piperidinium trifluoroacetate salt as the catalyst and toluene as the solvent appears to be critical for a successful annulation. We also demonstrated for the first time that microwave irradiation can accelerate aza-[3 + 3] annulation reactions. An attempt to expand the scope of enone aza-[3 + 3] annulation was made in the form of propyleine synthesis as a proof of concept. While the synthesis of an enone annulation precursor was successfully accomplished, the annulation itself proved to be challenging and was only modestly successful. Future investigations are underway to pursue alkaloid synthesis via enone aza-[3 + 3] annulation.

## Supporting Information

File 1 Experimental section.
