# Supplementary material for: Establishing the concept of aza-[3 + 3] annulations using enones as a key expansion of this unified strategy in alkaloid synthesis
Source: Beilstein J Org Chem. 2013 Jun 18;9:1170–8. doi: 10.3762/bjoc.9.131 (PMC3701412; doi:10.3762/bjoc.9.131)
Supplement: File 1 — Experimental section. [file Beilstein_J_Org_Chem-09-1170-s001.pdf]

Supporting Information

for

Establishing the concept of aza-[3 + 3] annulations using enones as a key expansion of this unified strategy in alkaloid synthesis

Aleksey I. Gerasyuto, Zhi-Xiong Ma, Grant S. Buchanan and Richard P. Hsung*

Address: Division of Pharmaceutical Sciences, School of Pharmacy, and Department of Chemistry, University of Wisconsin, Madison, WI 53705, USA

Email: Richard P. Hsung - rhsung@wisc.edu

Experimental section

General Experimental Information

All reactions were performed in flame-dried glassware under nitrogen atmosphere. Solvents were distilled prior to use. Reagents were used as purchased from Aldrich, Acros, Alfa Aesar, or TCI unless otherwise noted. Chromatographic separations were performed using Silicycle 43-60 Å SiO_2. ^{1}H and ^{13}C NMR spectra were obtained on Varian VI-400 and VI-500 spectrometers using $CDCl_3$ with TMS or residual solvent as standard unless otherwise noted. Melting points were determined using a Laboratory Devices MEL-TEMP and are uncorrected/calibrated. Infrared spectra were obtained on Bruker EQUINOX 55 FTIR. TLC analysis was performed using Aldrich 254 nm polyester-backed plates (60 Å, 250 µm) and visualized using UV and $KMnO_4$ stains. Low-resolution mass spectra were obtained using an Agilent 1100 series LS/MSD and are APCI. All spectral data obtained for new compounds are reported here.

Preparation of Enone 10:

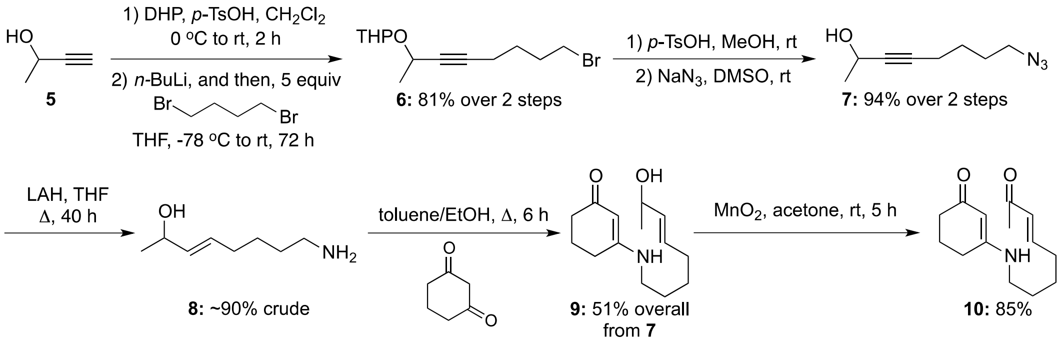

Bromide 6.

To a solution of the THP-protected alcohol prepared from 3-butyn-2-ol (**5**) in 93% yield via standard procedure (5.00 g, 32.42 mmol) in THF (40 mL) at −78 °C was added *n*-BuLi (1.6 M in hexanes, 25.0 mL, 40.53 mmol). The reaction mixture was stirred at this temperature for approximately 45 min, at which point 1,4-dibromobutane (19.2 mL, 162.2 mmol) was rapidly added. The resulting solution was allowed to warm to ambient temperature then stirred for 72 h at ambient temperature. The reaction mixture was quenched with H_2O (2 mL) and concentrated in vacuo. The crude residue was diluted with H_2O (40 mL) and the aqueous layer was extracted with CH_2Cl_2 (3 × 40 mL). The combined organic extracts were washed with brine (75 mL), dried over Na_2SO_4. 1,4-dibromobutane and solvents were removed under reduced pressure, to give a crude material that was then purified by Kugelrohr bulb-to-bulb distillation. After a small forerunning, bromide **6** (8.16 g, 87%) was retrieved [130–140 °C; 0.15 mmHg] as a mixture of two diastereomers (~6:1 ratio).

6: R_f = 0.45 (15% EtOAc in hexanes); *major isomer* ^{1}H NMR (500 MHz, $CDCl_3$) δ 1.43 (d, 3H, *J* = 7.0 Hz), 1.53-1.62 (m, 4H), 1.66 (quint, 2H, *J* = 7.5 Hz), 1.73-1.78 (m, 1H), 1.81-1.86 (m, 1H), 1.77 (quint,

2H, J = 7.5 Hz), 2.26 (td, 2H, J = 2.0, 7.0 Hz), 3.43 (t, 2H, J = 6.5 Hz), 3.49-3.54 (m, 1H), 3.82 (ddd, 1H, J = 3.5, 8.0, 11.5 Hz), 4.53 (qd, 1H, J = 2.0, 7.0 Hz), 4.92 (t, 1H, J = 3.0 Hz); IR (film) cm^{-1} 2941s, 2868s, 2362w, 2212m, 1728s, 1712s, 1676s, 1440s, 1116s; *minor isomer*: ^1H NMR (500 MHz, $CDCl_3$) δ 1.41 (d, 3H, J = 6.5 Hz), 1.53-1.62 (m, 4H), 1.63-1.72 (m, 2H), 1.73-1.78 (m, 1H), 1.81-1.86 (m, 1H), 1.92 (quint, 2H, J = 7.5 Hz), 2.27 (td, 2H, J = 2.0, 7.0 Hz), 3.43 (t, 2H, J = 6.5 Hz), 3.49-3.54 (m, 1H), 3.98 (ddd, 1H, J = 3.5, 8.0, 11.5 Hz), 4.47 (qd, 1H, J = 2.0, 6.5 Hz), 4.75 (t, 1H, J = 3.5 Hz).

Azido-alcohol 7.

In a 250 mL round bottom flask equipped with magnetic stir bar, bromide **6** (8.06 g, 27.87 mmol) was taken up in MeOH (75 mL) and *p*-TsOH (0.47 g, 2.4 mmol) was added in one portion. The solution was stirred at ambient temperature for 1.5 h, as TLC analysis [R_f = 0.16 (15% EtOAc in petroleum ether)] indicated complete consumption of the starting material. The solution was neutralized by addition of solid $NaHCO_3$ (0.60 g). The excess solid was filtered off through a fritted funnel and MeOH was removed in vacuo to give an alcohol that was used in the next step without further purification.

To a solution of crude alcohol (made from 8.06 g, 27.87 mmol **6**) in DMSO (90 mL) was added NaN_3 (3.08 g, 47.38 mmol). The reaction mixture was stirred for 2 h at ambient temperature (note TLC analysis is difficult due to similar R_f values), then H_2O (100 mL) was added and the aqueous layer was extracted with MTBE (4 × 100 mL). The combined organic extracts were washed with brine (75 mL), dried over Na_2SO_4, and concentrated to a crude residue, which was normally used without further purification. The material could be purified by filtration through a short silica gel column (MTBE, isocratic eluent), which then gave azido alcohol **7** (4.38 g, 94%) as a clear, colorless oil. **7**: ^1H NMR (500 MHz, $CDCl_3$) δ 1.19 (s, 1H, OH), 1.43 (d, 3H, J = 11.0 Hz), 1.55-1.65 (m, 2H), 1.67-1.78 (m, 2H), 2.27 (td, 2H, J = 3.5, 11.5 Hz), 3.31 (t, 2H, J = 6.6 Hz), 4.47-4.57 (m, 1H); mass spectrum (APCI): *m/e* (% relative intensity) 252 (100) $(M + H)^+$.

Vinylogous amide 9.

In a 1 liter three-neck round bottom flask equipped with thermometer was made a suspension of LAH (7.54 g, 198.79 mmol) in anhydrous THF (290 mL). To this suspension was added *slowly* (dropwise) a solution of azido alcohol **7** (5.54 g, 33.13 mmol) in THF (100 mL) while maintaining the internal temperature below 30 °C. An additional portion of THF (10 mL) was used to ensure complete salvation of the azido alcohol. When the bubbling of the reaction mixture slowed, the cool bath was replaced with an oil bath and a water-cooled condenser was fitted to the flask. The mixture was heated under reflux for

40 h, at which point the flask was removed from the heat bath and allowed to cool. The excess LAH was destroyed by the addition of saturated aqueous Na_2SO_4 (20 mL) added slowly due to a large exotherm. A white precipitate formed and the mixture was filtered, washing with MTBE (3 × 65 mL). The filtrate was then dried over Na_2SO_4, and concentrated to provide amino alcohol intermediate (4.27 g, 90%) as a pale yellow oil, which was used without further purification. ^{1}H NMR (500 MHz, $CDCl_3$) δ 1.25 (d, 3H, *J* = 10.5 Hz), 1.41-1.63 (br m, 7H), 2.04 (q, 2H, *J* = 10.5 Hz), 2.69 (t, 2H, *J* = 11.5 Hz), 4.26 (quint, 1H, *J* = 10.5 Hz), 5.48-5.68 (m, 2H).

A solution of amino alcohol intermediate (3.31 g, 23.11 mmol) and 1,3-cyclohexanedione in EtOH (7.5 mL) and toluene (75 mL) was prepared in a 250 mL round bottom flask. A water-cooled condenser was placed on the flask and the mixture was heated at 80 °C for 6–7 h, and then allowed to cool to ambient temperature. The solvents were removed and the crude material was purified via silica gel flash column chromatography (neutralized SiO_2 with 2% Et_3N in hexanes; 9–12% MeOH in CH_2Cl_2) to afford vinylogous amide **9** (2.83 g, 11.92 mmol, 52%) as a pale yellow solid.

9: R_f = 0.36 (1:7 MeOH in CH_2Cl_2); ^{1}H NMR (500 MHz, $CDCl_3$) δ 1.26 (d, 3H, *J* = 6.5 Hz), 1.45 (quint, 2H, *J* = 7.5 Hz), 1.57-1.63 (m, 2H), 1.97 (quint, 2H, *J* = 7.0 Hz), 2.07 (q, 2H, *J* = 7.0 Hz), 2.32 (t, 2H, *J* = 6.5 Hz), 2.33 (t, 2H, *J* = 6.5 Hz), 3.08 (q, 2H, *J* = 6.5 Hz), 4.28 (quint, 1H, *J* = 6.5 Hz), 4.52 (br s, 1H, NH), 5.13 (s, 1H), 5.55 (dd, 1H, *J* = 6.0, 15.5 Hz), 5.55 (dt, 1H, *J* = 6.0, 15.5 Hz); ^{13}C NMR (125 MHz, $CDCl_3$) δ 22.1, 23.7, 26.5, 27.9, 29.9, 31.5, 36.5, 42.8, 68.7, 96.7, 129.7, 135.5, 164.9, 197.5; IR (film cm^{-1}) 3256brm, 3070m, 2935m, 1536s, 1456m, 1364m, 1250s; mass spectrum (APCI): *m/e* (% relative intensity) 238 (85) $(M + H)^+$, 220 (100), 192 (10); HRMS (MALDI): m/e calcd for $C_{14}H_{23}NO_2Na^+$ 260.1626, found 260.1622.

Enone 10.

Vinylogous amide **9** (0.340 g, 1.43 mmol) was taken up in acetone (15 mL), and activated MnO_2 (88%, 3.23 g, 37.25 mmol) was added. The reaction mixture was stirred for 2 h. Occasionally, the allyl alcohol was slow to oxidize, so additional amounts of oxidant were added to ensure complete consumption of the starting material. After the reaction was deemed complete, the solid was filtered off using a 3 inch bed of Celite™ and washed with acetone. The filtrate was concentrate in vacuo and was then purified by silica gel flash column chromatography (9% MeOH in EtOAc) to yield enone **10** (0.219 g, 65%) as a reddish-yellow oil.

10: R_f = 0.45 (1:7 MeOH in CH_2Cl_2); ^{1}H NMR (500 MHz, $CDCl_3$) δ 1.51-1.58 (m, 2H), 1.60-1.66 (m, 2H), 1.98 (quint, 2H, *J* = 6.5 Hz), 2.25 (s, 3H), 2.26 (qd, 2H, *J* = 1.5, 7.0 Hz), 2.32 (t, 4H, *J* = 6.5 Hz),

3.10 (q, 2H, J = 6.5 Hz), 4.42 (br s, 1H, NH), 5.12 (s, 1H), 6.09 (dt, 1H, J = 1.5, 15.5 Hz), 6.77 (dt, 1H, J = 7.0, 15.5 Hz), 9.51 (d, 1H, J = 7.5 Hz); ^{13}C NMR (125 MHz, $CDCl_3$) δ 22.1, 25.6, 27.1, 28.1, 29.8, 32.1, 36.6, 42.7, 96.6, 131.7, 147.4, 164.9, 194.3, 198.7; IR (film) cm^{-1} 3256brm, 3070m, 2940m, 1673s, 1538s, 1466m, 1364m, 1250s, 1192s; mass spectrum (APCI): *m/e* (% relative intensity) 236 (100) $(M + H)^+$, 218 (15); HRMS (MALDI): m/e calcd for $C_{14}H_{21}NNaO_2Na^+$, 258.1470; found, 258.1468.

Intramolecular Aza-[3 + 3] annulation of enone 10.

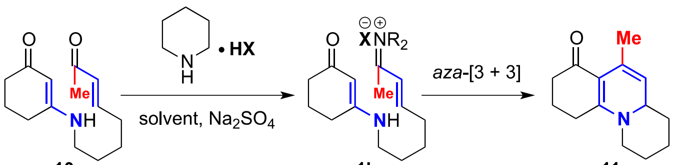

Procedure with conventional heating. In a flame-dried sealed tube was made a solution of enone **10** (23.5 mg, 0.100 mmol) in toluene (3.0 mL). To this solution was added piperidinium trifluoroacetate salt (9.9 mg, 0.05 mmol) and Na_2SO_4 (72 mg) and the reaction was capped with a rubber septum. The headspace was flushed then flushed with a stream of N_2. The rubber septum was removed and the tube was quickly capped and then heated at 150 °C for 3 h. The reaction mixture was allowed to cool to room temperature, and it was diluted with EtOAc (5 mL), then washed with H_2O (5 mL), and saturated aqueous $NaHCO_3$ (5 mL). The aqueous washes were back-extracted with EtOAc (2 × 5 mL), and the combined organic extracts were washed with brine (10 mL), dried over Na_2SO_4 and concentrated to a crude semisolid, which was purified by silica gel flash column chromatography (9–12% MeOH in CH_2Cl_2) to afford aza-tricycle **11** (19 mg, 87%) as a yellow oil, which solidified upon placement under high-vacuum.

Procedure with microwave irradiation. Mixture of enone **10** (0.0759 g, 0.322 mmol), piperidinium trifluoroacetate salt (0.019 g, 0.095 mmol, 30 mol %) and powdered Na_2SO_4 (~0.10 g) in EtOAc (3 mL) and toluene (4.5 mL) was microwaved on high (2 × 15 min) in a sealed tube. Additional portion of piperidinium trifluoroacetate (0.037 g, 0.186 mmol, 60 mol %) was added to the reaction mixture and microwave irradiation continued for another 3 × 20 min. After cooling the reaction to rt it was diluted with EtOAc (4 mL) and washed with H_2O (2 × 10 mL). Aqueous washes were back-extracted with EtOAc (10 mL). Combined organic phase were washed with brine (10 mL) and dried over Na_2SO_4.

Evaporation of the solvent afforded aza-tricycle **11** (0.048 g, 68%) as a dark yellow oil. Product was purified by silica gel column chromatography [gradient eluent: CH_2Cl_2 to CH_2Cl_2/MeOH (10:1)] to afford pure annulation product **11** (0.0337 g, 54%) as yellow oil, which slowly solidified upon keeping on high vacuum.

11: R_f = 0.45 (1:7 MeOH in CH_2Cl_2); ^{1}H NMR (500 MHz, $CDCl_3$) δ 1.46 (br d, 1H, J = 12.5 Hz), 1.75 (m, 2H), 1.87-1.93 (m, 3H), 2.12 (s, 3H), 2.27 (t, 2H, J = 7.5 Hz), 2.38 (ddd, 1H, J = 5.5, 8.0, 17.0 Hz), 2.51 (dt, 1H, J = 6.0, 17.0 Hz), 2.77 (td, 1H, J = 2.5, 12.5 Hz), 3.96-4.02 (m, 2H), 4.73 (dd, 1H, J = 1.5, 3.5 Hz); ^{13}C NMR (125 MHz, $CDCl_3$) δ 20.8, 22.6, 25.1, 26.9, 27.1, 33.7, 37.3, 48.7, 60.4, 107.9, 113.9, 132.2, 160.7, 192.8; mass spectrum (APCI): *m/e* (% relative intensity) 218 (100) $(M + H)^+$; HRMS (MALDI): m/e calcd for $C_{14}H_{19}NONa^+$, 240.1364; found, 240.1365.

Studies toward propyleine:

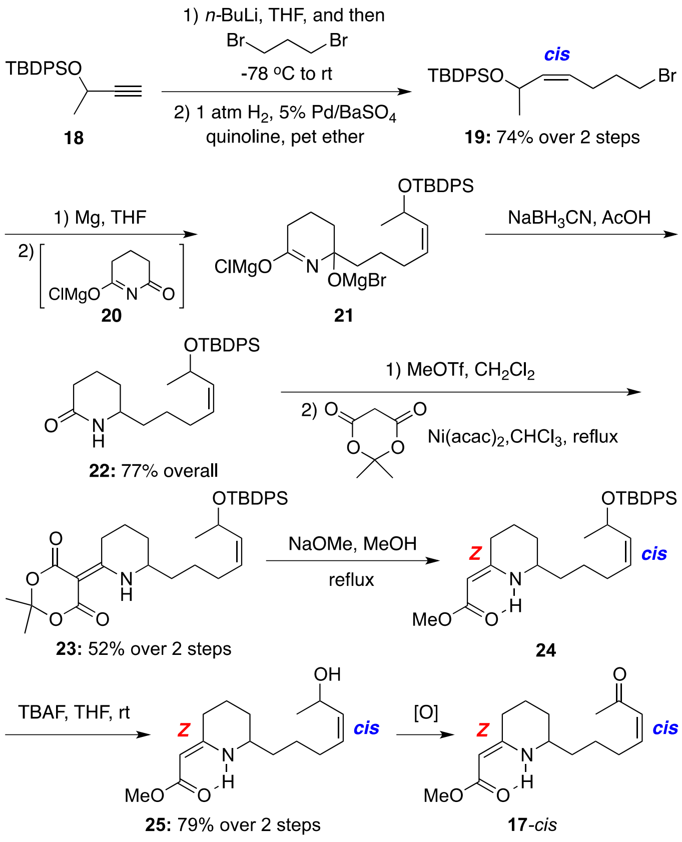

Bromide 19.

To a solution of 3-butyne-2-ol (16.5 mL, 0.210 mol) and imidazole (12.85 g, 0.188 mol) in THF (80 mL) was added solution of TBDPSCl (51.87 g, 0.188 mol) in THF (50 mL) dropwise over 15–20 min. The reaction was stirred at rt for 3 h before H_2O (100 mL) was added. The layers were separated and the aqueous phase was back-extracted with CH_2Cl_2 (2 × 50 mL). The combined organic layers were washed with sat aq NaCl (100 mL) and dried over Na_2SO_4. Solvents were removed under reduced pressure, and the crude residue was purified by vacuum distillation. Alkyne **18** (48.37 g, 83%) was collected at 130-135 °C (0.3 mmHg) as a colorless oil.

18: ^{1}H NMR (500 MHz, $CDCl_3$): δ 1.08 (s, 9 H), 1.39 (d, 3 H, *J* = 6.5 Hz), 2.33 (d, 2 H, *J* = 2.0 Hz), 4.45 (qd, 1 H, *J* = 6.5, 2.0 Hz), 7.36 – 7.40 (m, 4 H), 7.41 – 7.45 (m, 2 H), 7.68 (dd, 2 H, *J* = 7.5, 1.5 Hz), 7.75 (dd, 2 H, *J* = 8.0, 1.5 Hz).

To a solution of alkyne **18** (48.37 g, 0.157 mol) in THF (200 mL) was added *n*-BuLi (2.5 M in hexanes, 80.0 mL, 0.200 mol) at −78 °C. The reaction was stirred at this temperature for 20 min before 1,3-dibromopropane (80.0 mL, 0.785 mol) was added in one portion. The resulting mixture was allowed to warm up to rt and was stirred for 72 h before it was quenched with H_2O (100 mL). The aqueous layer was back-extracted with MTBE (2 × 50 mL). The combined organic layers were washed with sat aq NaCl (100 mL) and dried over Na_2SO_4. The solvents and excess of 1,3-dibromopropane were removed under reduced pressure and the crude residue was purified via Kugelrohr distillation. The bromide intermediate (59.63 g, 88%) was collected at 190–195 °C (0.3 mmHg) as pale yellow oil. ^{1}H NMR (500 MHz, $CDCl_3$): δ 1.06 (s, 9 H), 1.39 (d, 3 H, *J* = 6.5 Hz), 1.87 (quint, 2 H, *J* = 7.0 Hz), 2.25 (td, 2 H, *J* = 7.0, 2.0 Hz), 3.36 (t, 2 H, *J* = 6.5 Hz), 4.47 (qt, 1 H, *J* = 6.5, 2.0 Hz), 7.36-7.44 (m, 6 H), 7.69 (dd, 2 H, *J* = 7.5, 1.5 Hz), 7.74 (dd, 2 H, *J* = 8.0, 1.5 Hz); ^{13}C NMR (125 MHz, $CDCl_3$) δ 17.6, 19.4, 25.6, 27.1, 31.5, 32.6, 60.3, 82.2, 84.0, 127.6, 127.8, 129.8, 129.9, 134.1, 134.2, 136.0, 136.2.

A suspension of 5% Pd/$BaSO_4$ catalyst (2.04 g) in petroleum ether (120 mL) was prereduced via hydrogenation until the color changed from complete brown to black (within ~ 20–30 min). After which, quinoline (2.0 mL) was added to the above suspension and the resulting mixture was stirred for 15 min before a solution of the bromide made above (24.2 g, 56.35 mmol) in petroleum ether (120 mL) was added. The reaction mixture was hydrogenated with a H_2-balloon for 5 h until complete consumption of

the starting material was observed through ^{1}H NMR. The excess solvent was removed under reduced pressure and the crude residue was purified via Kugelrohr distillation. Bromide **19** (23.0 g, 95%) was collected at 165–170 °C (0.1 mmHg) as a pale yellow oil. **19**: ^{1}H NMR (300 MHz, $CDCl_3$) δ 1.04 (s, 9 H), 1.18 (d, 3 H, *J* = 6.3 Hz), 1.59-1.86 (m, 4 H), 3.17 (t, 2 H, *J* = 6.6 Hz), 4.57 (quint, 1 H, *J* = 6.6 Hz), 5.10 (dt, 1 H, *J* = 11.1, 6.6 Hz), 5.55 (dd, 1 H, *J* = 11.1, 8.7 Hz), 7.32-7.45 (m, 6 H), 7.65-7.70 (m, 4 H); ^{13}C NMR (75 MHz, $CDCl_3$) δ 19.4, 24.9, 26.1, 27.1, 32.6, 33.3, 66.0, 126.3, 127.6, 127.7, 129.68, 129.73, 134.4, 134.7, 136.0, 136.1, 136.3.

Lactam 22.

Preparation of 20. To a solution of glutarimide (2.85 g, 25.20 mmol) in THF (80 mL) was added CH_3MgCl (3 M in THF, 9.0 mL, 27.0 mmol) dropwise at 0 °C. The reaction mixture was allowed to warm up to rt and was stirred for 30 min and then cooled back down to 0 °C.

Formation of 21. A warm solution of Grignard reagent (35–40 °C to prevent precipitation) in THF (30 mL) prepared from bromide **19** (16.23 g, 37.60 mmol) and Mg (1.04 g, 43.33 mmol) through activation using dibromoethane (0.50 mL, 5.80 mmol) was added via a cannula.

The resulting reaction mixture was stirred for 12 h at rt before $NaBH_3CN$ (1.74 g, 27.69 mmol) and HOAc (7.5 mL) were added successively. After stirring at ambient temperature for 45 min, reaction was quenched with sat aq $NaHCO_3$ solution (80 mL). After layer separation, the organic phase was washed with an additional portion of $NaHCO_3$ solution (80 mL). Aqueous washes were back-extracted with EtOAc (3 × 80 mL). The combined organic layers were washed with sat aq NaCl (2 × 50 mL) and dried over Na_2SO_4. After solvent removal under reduced pressure, the crude residue was purified through two silica gel columns (1st: 0–50% acetone in EtOAc; 2nd: 7–10% MeOH in CH_2Cl_2) to afford lactam **22** (8.70 g, 77%) as a thick colorless oil.

22: ^{1}H NMR (300 MHz, $CDCl_3$) δ 0.95-1.09 (m, 1H), 1.07 (s, 9H), 1.13-1.27 (m, 6H), 1.17 (d, 3H, *J* = 6.0 Hz), 1.57-1.71 (m, 3H), 1.78-1.89 (m, 2H), 2.22 (td, 1H, *J* = 6.0, 3.0 Hz), 2.31-2.62 (m, 1H), 3.18-3.26 (m, 1H), 4.54 (quint, 1H, *J* = 6.3 Hz), 5.12 (dt, 1H, *J* = 12.0, 7.2 Hz), 5.53 (dd, 1H, *J* = 12.0, 8.4 Hz), 5.66 (brs, 1H, NH), 7.33-7.46 (m, 6H), 7.63-7.71 (m, 4H).

Meldrum's Acid Derivative 23.

To a solution of lactam **22** (1.10 g, 2.45 mmol) in CH_2Cl_2 (15 mL) was added freshly distilled MeOTf (0.35 mL, 3.09 mmol). The reaction mixture was stirred at ambient temperature for 8 h before the reaction mixture was treated with cold 5% aq Na_2CO_3 (20 mL). After separation of the layers, the aqueous phase was extracted with CH_2Cl_2 (2 × 10 mL). Combined organics were washed with sat aq NaCl (15 mL), and dried over Na_2SO_4. Removal of the excess solvent gave the desired *O*-methyl lactim ether as a colorless oil, which was used for the next step without further purification.

To a solution of the above *O*-methyl lactim ether in $CHCl_3$ (15 mL) were successively added Meldrum's acid (0.350 g, 2.43 mmol) and Ni(acac)$_2$ catalyst (ca. 25.0 mg). The reaction mixture was heated in a sealed tube at 70–80 °C for 18 h under N_2 atmosphere. Upon solvent removal under reduced pressure, the crude residue was purified by silica gel flash column chromatography (20–50% EtOAc in hexanes) to give Meldrum's acid derivative **23** (0.74 g, 52% over 2 steps) as a colorless oil.

23: ^{1}H NMR (500 MHz, $CDCl_3$) δ 1.03 (s, 9H), 1.17 (d, 3H, *J* = 6.0 Hz), 1.22-1.41 (m, 5H), 1.60-1.71 (m, 3H), 1.67 (s, 3H), 1.68 (s, 3H), 1.86-1.91 (m, 2H), 2.92-2.99 (m, 1H), 3.31-3.36 (m, 2H), 4.52-4.58 (m, 1H), 5.15 (dt, 1H, *J* = 11.0, 7.0 Hz), 5.54 (t, 1H, *J* = 11.0 Hz), 7.33-7.44 (m, 6H), 7.65-7.69 (m, 4H), 11.62 (brs, 1H, NH).

Alcohol 25.

To a solution of MeONa prepared by dissolving Na metal (0.640 g, 27.82 mmol) in anhydrous MeOH (35 mL) was added a solution of compound **23** (3.22 g, 5.59 mmol) in MeOH (35 mL). The reaction mixture was heated under reflux overnight. After cooling the reaction mixture to rt, MeOH was evaporated and H_2O (60 mL) was added to the crude residue, and subsequently, aq HCl (1 M) was also added dropwise to adjust the pH to 5–6. The resulting mixture was extracted with CH_2Cl_3 (3 × 70 mL), the combined organic layers were washed with sat aq NaCl (2 × 25 mL) and dried over Na_2SO_4. Upon removal of excess solvents under reduced pressure, the crude vinylogous urethane **24** was submitted to the next step without further purification.

To a solution of crude vinylogous urethane **24** in THF (30 mL) was added TBAF solution (1 M in THF, 6.4 mL, 6.4 mmol). Reaction mixture was stirred at rt for 2 h before another portion of TBAF solution (5.0 mL, 5.0 mmol) was added. After complete consumption of starting material as observed by TLC

analysis THF was removed under reduced pressure and the residue was purified by silica gel column chromatography (50% EtOAc in hexanes) to yield allyl alcohol **25** (1.18 g, 79% over 2 steps) as a colorless oil. ^{13}C NMR spectra confirmed the presence of two diatereomers in 1:1 ratio.

25: ^{1}H NMR (500 MHz, $CDCl_3$) δ 1.25 (d, 3H, *J* = 6.0 Hz), 1.36 (qd, 1H, *J* = 10.5, 3.5 Hz), 1.46-1.66 (m, 5H), 1.75-1.81 (m, 1H), 1.87-1.94 (m, 1H), 2.03-2.14 (m, 1H), 2.16-2.26 (m, 1H), 2.31-2.35 (m, 2H), 3.27 (brs, 1H), 3.61 (s, 3H), 4.37 (s, 1H), 4.64 (brs, 1H), 5.40-5.48 (m, 2H), 8.85 (brs, 1H, NH) 8.89 (brs, 1H, NH); ^{13}C NMR (125 MHz, $CDCl_3$) δ 19.7, 23.7, 23.8, 25.76, 25.84, 27.1, 27.6, 29.4, 29.5, 29.6, 36.5, 36.8, 50.1, 50.2, 51.6, 52.0, 63.7, 63.9, 79.88, 79.92, 130.2, 130.4, 134.8, 134.9, 163.0, 163.1, 171.4; mass spect (APCI): *m/e* (% relative intensity) 268 (100) $(M + H)^+$; HRMS (MALDI): m/e calcd for $C_{15}H_{25}NO_3Na^+$, 290.1732; found, 290.1730.

Enone 17-*cis*.

To the pyr·SO_3 complex (0.273 g, 1.72 mmol) were added pyridine (0.14 mL, 1.75 mmol) and DMSO (0.30 mL, 4.22 mmol) successively at rt. The resulting suspension was stirred at rt for 15 min before CH_2Cl_2 (2 mL) was added, and then the mixture was cooled to 0 °C. A precooled solution of alcohol **25** (0.2293 g, 0.86 mmol), diisopropylethylamine (0.50 mL, 3.01 mmol), and DMSO (0.30 mL, 4.22 mmol) in CH_2Cl_2 (5 mL) was added to the suspension dropwise. The reaction mixture was stirred at 0 °C for 1 h before H_2O (6 mL) was added. After the layers had been separated, the organic phase was washed once with H_2O (6 mL). The combined aqueous washes were back-extracted with CH_2Cl_2 (2 × 6 mL). The combined organic phases were washed with 1.0% aq HCl (2 × 7 mL), aq $NaHCO_3$ (6 mL), sat aq NaCl (6 mL) and dried over Na_2SO_4. Removal of the excess solvent gave enone **17-*cis*** (0.1938 g, 85%) as yellow oil. This was used for the annulation reaction without further purification.

17-*cis*: ^{1}H NMR (500 MHz, $CDCl_3$) δ 1.36 (dddd, 1H, *J* = 13.5, 11.5, 10.0, 3.5 Hz), 1.49-1.63 (m, 5H), 1.75-1.80 (m, 1H), 1.88-1.93 (m, 1H), 2.21 (s, 3H), 2.32 (q, 2H, *J* = 5.5 Hz), 2.65 (qd, 2H, *J* = 7.0, 1.0 Hz), 3.25-3.29 (m, 1H), 3.62 (s, 3H), 4.36 (s, 1H), 6.06 (dt, 1H, *J* = 11.5, 7.5 Hz), 6.16 (brd, 1H, *J* = 11.5 Hz), 8.81 (brs, 1H, NH); ^{13}C NMR (125 MHz, $CDCl_3$) δ 19.7, 25.2, 29.2, 29.38, 29.40, 31.8, 36.9, 50.0, 51.9, 79.9, 127.6, 147.8, 163.0, 171.2, 199.4; mass spect (APCI): *m/e* (% relative intensity) 266 (100) $(M + H)^+$; HRMS (MALDI): m/e calcd for $C_{15}H_{23}NO_3Na^+$, 288.1576; found, 288.1577.

Enone 17-*cis* isomerization.

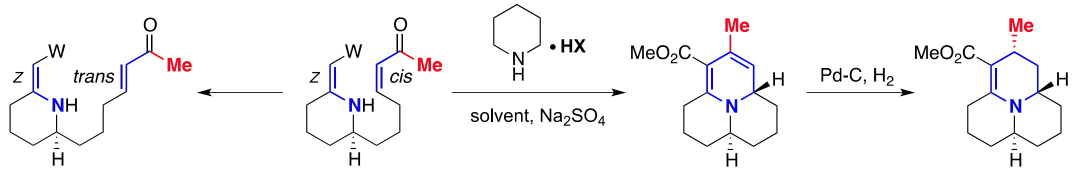

To a solution of freshly prepared enone **17**-*cis* (30.8 mg, 0.116 mmol) in freshly distilled EtOAc (4 mL) under N_2 atmosphere were added powdered Na_2SO_4 (~100.0 mg) and piperidinium trifluoroacetate (23.0 mg, 0.115 mmol). The reaction mixture was heated at 55 °C until complete consumption of starting material (~12–14 h) as indicated by TLC analysis (**17**-*trans* has lower R_f -value). Then the excess solvent was evaporated under reduced pressure. The crude residue was submitted to silica gel flash column chromatography (50% EtOAc in hexanes). Enone **17**-*trans* (25.0 mg, 85%) was obtained as a yellow oil.

17-*trans*: ^{1}H NMR (400 MHz, $CDCl_3$) δ 1.34-1.40 (m, 1H), 1.49-1.70 (m, 5H), 1.75-1.83 (m, 1H), 1.89-1.93 (m, 1H), 2.25-2.29 (m, 2H), 2.25 (s, 3H), 2.30-2.35 (m, 2H), 3.27 (sextet, 1H, J = 5.6 Hz), 3.62 (s, 3H), 4.38 (s, 1H), 6.09 (dt, 1H, J = 16.0, 1.6 Hz), 6.78 (dt, 1H, J = 16.0, 6.8 Hz), 8.84 (brs, 1H, NH).

Cycloadduct 16.

To a solution of freshly prepared enone **17**-*cis* (193.8 mg, 0.730 mmol) in dry degassed toluene (7 mL) under N_2 atmosphere were added powdered Na_2SO_4 (~100.0 mg) and piperidinium trifluoroacetate (150.0 mg, 0.750 mmol). The reaction mixture was heated at 100 °C for 5 h before the excess solvent was evaporated under reduced pressure. The crude residue was submitted to flash column chromatography using silica gel deactivated with NEt_3 (2% in EtOAc:hexanes 9:1) eluted with 10% EtOAc in hexanes). Cycloadduct **16** (60.3 mg, 34% yield) was obtained as a very air-sensitive yellow oil with significant contamination according to ^{1}H NMR analysis. Representative data: ^{1}H NMR (500 MHz, $CDCl_3$) δ 1.96 (s, 3H), 4.55 (s, 1H). Hydrogenation of **16** using standard conditions of Pd/C and 1 atm H_2 led to an inseparable diastereomeric mixture [~3:1] of **26**. Representative data: *major product* ^{1}H NMR (500 MHz, $CDCl_3$) δ 1.02 (d, 3H, J = 6.5 Hz), 3.14-3.24 (m, 2H), 3.65 (s, 3H); *minor product* ^{1}H NMR (500 MHz, $CDCl_3$) δ 1.07 (d, 3H, J = 7.5 Hz), 3.62 (s, 3H); mass spect (APCI): *m/e* (% relative intensity) 250 (100) $(M + H)^+$, 218 (10).
